# Developmental Odontogenic Cysts of Jaws: A Clinical Study of 245 Cases

**DOI:** 10.5681/joddd.2009.015

**Published:** 2009-06-05

**Authors:** Javad Yazdani, Shiva Solahaye Kahnamouii

**Affiliations:** ^1^Assistant Professor, Department of Oral and Maxillofacial Surgery, Faculty of Dentistry, Tabriz University of Medical Sciences, Tabriz, Iran; ^2^Dental Student, Faculty of Dentistry, Tabriz University of Medical Sciences, Tabriz Iran

**Keywords:** Developmental cyst, oral cyst, dentigerous cyst, odontogenic keratocyst

## Abstract

**Background and aims:**

The aim of this study was to investigate the relative frequency of developmental odontogenic cysts in an Iranian population.

**Materials and methods:**

In this study 245 cysts from both jaws, treated in the Faculty of Dentistry at Tabriz University of Medical Sciences during a 10-year period from 1998 to 2008, were analyzed in order to evaluate the incidence of such cysts. We had permission from all the patients. Case histories of 65% of male and 35% of female patients were analyzed. The age of the patients varied from 14 to 64 years, with an average of 33.21 ± 10.89.

**Results:**

In this 10-year study of odontogenic cysts, 97 cases were developmental odontogenic cysts with the following inci-dence: dentigerous cyst, 44%; odontogenic keratocyst, 36%; primordial cyst, 9%; Gorlin cyst, 2%; lateral periodontal cyst, 3%; eruption cyst, 3%; and gingival cyst, 3% (adults 2%, infants 1%). A total of 60% of the cysts were found in the mandible and 40% in the maxilla. Regarding the mandible, the molar region was involved in 47% of the cases, premolar region in 33% and anterior region in 20% (total = 100%). Regarding the maxilla, the canine-to-canine region was involved in 52% of the cases, premolar region in 20% and molar region in 28% (total = 100%).

**Conclusion:**

An important finding in this study was the fact that 39% of the jaw cysts were developmental odontogenic cysts and the most common developmental odontogenic cysts were dentigerous cyst and OKC (odontogenic keratocyst).

## Introduction

Odontogenic cysts are the most common form of cystic lesions affecting the maxillofacial region.

They are classified traditionally into a developmental group, including keratocysts and dentigerous cysts, and an inflammatory group, including radicular cysts. ^1 , 2 , 3^ A cyst is a pathologic cavity (often fluidfilled) which is lined by epithelium. A number of different developmental cysts of the head and neck have been described. ^4^

With rare exceptions, epithelium-lined cysts in bone are seen only in the jaws. Other than a few cysts which may result from the inclusion of epithelium along embryonic lines of fusion, most jaw cysts are lined by epithelium which is derived from odontogenic epithelium. These are referred to as odontogenic cysts.

Odontogenic cysts are sub-classified as developmental or inflammatory in origin. Developmental cysts are of unknown origin, but they do not appear to be the result of an inflammatory reaction. Inflammatory cysts are the result of inflammation. The present categories of odontogenic cysts are based upon the classification of 1992 World Health Organization (WHO) (Table 1).^5,6^

**Table 1:** Classification of odontogenic cysts

Type	Odontogenic Cysts
Developmental	Dentigerous cyst Eruption cyst Odontogenic keratocyst Orthokeratinized odontogenic cyst Gingival (alveolar) cyst of the newborn Gingival cyst of the adult Lateral periodontal cyst Calcifying odontogenic cyst Glandular odontogenic cyst
Inflammatory	Periapical (radicular) cyst Residual periapical (radicular) cyst Buccal bifurcation cyst

Developmental cysts are usually asymptomatic, but have the potential to become extremely large and cause cortical expansion and erosion.^7^ Some cysts can be aggressive, demolishing the jaws or can be frequently recurrent.^8^There are many studies which have reported the relative frequency of developmental odontogenic jaw cysts, but neither of them is related to Iranian population. The purpose of this study was to investigate the relative frequency of developmental odontogenic cysts in an Iranian population.

## Materials and Methods

In this descriptive study, we analyzed 245 odontogenic cysts from 245 patients, treated in the Faculty of Dentistry, Department of Oral and Maxillofacial Surgery at Tabriz University of Medical Sciences, between 1998 and 2008. We obtained written consent forms from all the patients. Developmental odontogenic cysts data were retrieved from case notes, imaging, histopathology records and follow-up reports. In every case the following information was obtained: age, gender, location of the lesion, surgical treatment, and histopathologic diagnosis. Descriptive analysis was used for the categorical variables, central tendency measures, and dispersion of continuous variables with SPSS Software, Version 15.

## Results

The subjects were 14 to 64 years of age, with a mean of 33.21 ± 10.89 years. Male subjects outnumbered female subjects with a male-to-female ratio of 1.85:1. In this 10-year study of odontogenic cysts, 97 cases were developmental odontogenic cysts with the following incidence:

Dentigerous cyst, 44%; odontogenic keratocyst, 36%; primordial cyst, 9%; Gorlin cyst, 2%; lateral periodontal cyst, 3%; eruption cyst, 3%; gingival cyst, 3% (adults 2%, infants 1%) .

A total of 60% of the cysts were in the mandible and 40% in the maxilla. Regarding the mandible, the molar region was involved in 47% of the cases, premolar region in 33%, and anterior region in 20% (total = 100%).

Regarding the maxilla, the canine-to-canine region was involved in 52% of the cases, premolar region in 20%, and molar region in 28% (total = 100%).

## Discussion

The present study was a ten-year-old study to evaluate the prevalence of developmental odontogenic cysts in Tabriz Faculty of Dentistry. In this study 39% of the cysts were developmental odontogenic cysts, 44% of which were dentigerous, 36% were keratocysts, and 9% were primordial.

Mosqueda-Taylor et al^9^ analyzed 85 cases of odontogenic cysts in Mexico and reported that dentigerous cysts and keratocysts had a higher incidence after inflammatory cyst, coincident with the results of our study.

In 2000, Ledesma-Montes et al^10^ studied 3004 odontogenic cysts; 38.8% of the cases were periapical cysts, 35.5% were dentigerous, and 18.8% were OKC.

In another study in 1994 in Nigeria, Ogunlewe et al^11^ evaluated 126 cases of jaw cysts in Lagus University; 57.14% of the cases were developmental odontogenic cysts and 22.22% were dentigerous cysts.

Koseoglu et al^12^ and Oji^13^ reported different prevalence rates regarding dentigerous cysts and keratocysts, but the case series included only 90 and 20 patients, respectively.

Daley et al^14^ in 1994 in Canada reported that radicular cysts are the most common, and dentigerous cysts and keratocysts with 24.8% and 4.88% of the cases rank the second and third, respectively.

Eshghyar and Jalayer Naderi^15^ studied 1888 cases in Iran and reported that 736 cases were developmental odontogenic cysts, 51% were dentigerous cysts, 36% were keratocysts, 6% were Gorlin cyst, 3% were primordial cyst, 2% were developmental lateral periodontal cyst, 1% was eruption cysts, and 1% was adult gingival cyst.

Evaluation of the results of the above-mentioned studies shows that radicular cyst is the most common odontogenic cyst, coincident with the prevalence of dental caries.

Dentigerous cysts and keratocysts were the most common developmental odontogenic cysts in all the studies, which is consistent with the results of the present study.
